# 505 Risk Stratification by TBSA for Acute Kidney Injury in Burn Patients Following Combination Antibiotic Therapy

**DOI:** 10.1093/jbcr/irad045.102

**Published:** 2023-05-15

**Authors:** Kimberley Brondeel, Alen Palackic, Amina El Ayadi, Steven Wolf, Jong Lee, Juquan Song, Georgiy Golovko

**Affiliations:** University of Texas Medical Branch, Galveston, Texas; University of Texas Medical Branch at Galveston, Galveston, Texas; The University of Texas Medical Branch, Galveston, Texas; The University of Texas Medical Branch, Galveston, Texas; The University of Texas Medical Branch, Galveston, Texas; The University of Texas Medical Branch, Galveston, Texas; The University of Texas Medical Branch, Galveston, Texas; University of Texas Medical Branch, Galveston, Texas; University of Texas Medical Branch at Galveston, Galveston, Texas; The University of Texas Medical Branch, Galveston, Texas; The University of Texas Medical Branch, Galveston, Texas; The University of Texas Medical Branch, Galveston, Texas; The University of Texas Medical Branch, Galveston, Texas; The University of Texas Medical Branch, Galveston, Texas; University of Texas Medical Branch, Galveston, Texas; University of Texas Medical Branch at Galveston, Galveston, Texas; The University of Texas Medical Branch, Galveston, Texas; The University of Texas Medical Branch, Galveston, Texas; The University of Texas Medical Branch, Galveston, Texas; The University of Texas Medical Branch, Galveston, Texas; The University of Texas Medical Branch, Galveston, Texas; University of Texas Medical Branch, Galveston, Texas; University of Texas Medical Branch at Galveston, Galveston, Texas; The University of Texas Medical Branch, Galveston, Texas; The University of Texas Medical Branch, Galveston, Texas; The University of Texas Medical Branch, Galveston, Texas; The University of Texas Medical Branch, Galveston, Texas; The University of Texas Medical Branch, Galveston, Texas; University of Texas Medical Branch, Galveston, Texas; University of Texas Medical Branch at Galveston, Galveston, Texas; The University of Texas Medical Branch, Galveston, Texas; The University of Texas Medical Branch, Galveston, Texas; The University of Texas Medical Branch, Galveston, Texas; The University of Texas Medical Branch, Galveston, Texas; The University of Texas Medical Branch, Galveston, Texas; University of Texas Medical Branch, Galveston, Texas; University of Texas Medical Branch at Galveston, Galveston, Texas; The University of Texas Medical Branch, Galveston, Texas; The University of Texas Medical Branch, Galveston, Texas; The University of Texas Medical Branch, Galveston, Texas; The University of Texas Medical Branch, Galveston, Texas; The University of Texas Medical Branch, Galveston, Texas; University of Texas Medical Branch, Galveston, Texas; University of Texas Medical Branch at Galveston, Galveston, Texas; The University of Texas Medical Branch, Galveston, Texas; The University of Texas Medical Branch, Galveston, Texas; The University of Texas Medical Branch, Galveston, Texas; The University of Texas Medical Branch, Galveston, Texas; The University of Texas Medical Branch, Galveston, Texas

## Abstract

**Introduction:**

Burn patients are susceptible to infections and are often treated with broad-spectrum antibiotics. Vancomycin is a common antibiotic administered alone, or in combination with piperacillin-tazobactam (Pip/Tazo). This combination therapy was associated with increased rates of acute kidney injury (AKI) in burn patients when compared to Vancomycin alone or in combination with other antibiotics. However, recent studies failed to demonstrate these findings. We sought to determine the degree of nephrotoxicity of Vancomycin, Pip/Tazo, and their combination, with burn severity.

**Methods:**

A retrospective, multi-institutional study was performed utilizing TriNetX Global Health Research Network that encompasses de-identified patient records from 55 health care organization across the US. We identified the population of burn patients between 2000-2020 and stratified them into 3 groups by TBSA < 10%, 10-39%, or 40%+. Patients were further stratified into cohorts by administration of broad-spectrum antibiotics within 3 months of their burn: Vancomycin, Pip/Tazo alone, in combination (Combo), or neither. Cohorts were propensity score matched for age at index, gender, race, ethnicity, and pre-existing conditions that predispose to AKI. We explored patient distribution and conducted a comparative outcome analysis to determine the risk of developing AKI in 7- and 90-day outcome windows.

**Results:**

Vancomycin and Pip/Tazo were compared to control groups and showed increased risk of AKI in all TBSA groups and time outcomes(p< 0.0001). Before stratifying by TBSA, all burn patients administered combination antibiotics showed increased risk of AKI when compared to control, Vancomycin alone, and Pip/Tazo. In patients under < 10% TBSA, combo antibiotics increased AKI incidence in both 7- and 90-days when compared to all other groups. In the 10-39% TBSA group, combination therapy increased the risk of AKI when compared to Pip/Tazo at 7-days. Increased risk of AKI occurred in the combo group when compared to both Vancomycin and Pip/Tazo in 90-days. In the 40%+ TBSA cohort, combination antibiotics did not increase the incidence of AKI in either time period or when compared to Vancomycin and Pip/Tazo. The results of the comparative analysis are included in Table 1.

**Conclusions:**

Broad-spectrum antibiotics use in burn patients is associated with increased risk of AKI, this risk is higher with Vancomycin and Piperacillin/Tazobactam combination. Combo therapy increased AKI in < 10% and 10-39% but not 40%+ TBSA. These findings can be used to monitor for early manifestations of AKI when using broad-spectrum antibiotics.

**Applicability of Research to Practice:**

The decision to initiate antimicrobial therapy is crucial in burn patients as they are already at risk for AKI. TBSA can be a useful tool for guiding risk stratification.

